# Targeting lon protease to inhibit persister cell formation in *Salmonella* Typhimurium: a drug repositioning approach

**DOI:** 10.3389/fcimb.2024.1427312

**Published:** 2024-09-05

**Authors:** Negar Narimisa, Shabnam Razavi, Amin Khoshbayan, Sajjad Gharaghani, Faramarz Masjedian Jazi

**Affiliations:** ^1^ Microbial Biotechnology Research Center, Iran University of Medical Sciences, Tehran, Iran; ^2^ Department of Microbiology, School of Medicine, Iran University of Medical Sciences, Tehran, Iran; ^3^ Laboratory of Bioinformatics and Drug Design (LBD), Institute of Biochemistry and Biophysics, University of Tehran, Tehran, Iran

**Keywords:** *Salmonella*, toxin-antitoxin systems, lon protease, molecular docking, molecular dynamics simulation, diosmin, nafcillin

## Abstract

**Objective:**

Persister cells are a specific subset of bacteria capable of surviving exposure to lethal doses of antibiotics, leading to antibiotic therapy failures and infection relapses. This research explores the utilization of drug repositioning to target the Lon protease in *Salmonella* Typhimurium.

**Method:**

In this study, FDA-approved drugs sourced from the Drug Bank database were screened to identify existing pharmaceuticals with the potential to combat the Lon protease. The formation of persister cells in the presence of antibiotics, as well as the combination of antibiotics with potential Lon protease inhibitors, was examined. Furthermore, the expression of type II toxin-antitoxin system genes was analyzed to enhance our comprehension of the inhibitors’ effects.

**Result:**

Molecular docking analysis revealed that Diosmin and Nafcillin exhibited strong binding affinity to the Lon protease. Molecular dynamics simulation trajectories analysis demonstrated that the interaction of these ligands with the enzyme did not induce instability; rather, the enzyme’s structure remained stable. Combinations of ceftazidime and ciprofloxacin with either Nafcillin or Diosmin led to significant reductions in bacterial cell counts. Furthermore, the effectiveness of these combinations, when compared to antibiotics alone, highlighted the substantial impact of Nafcillin and Diosmin in reducing type II TA system gene expression.

**Conclusion:**

These findings suggest promising prospects for developing novel therapeutic approaches targeting persister cells to mitigate treatment failures in *Salmonella* infections.

## Introduction

Bacterial pathogens have developed mechanisms to evade antibiotic treatment, resulting in treatment failures and recurring infections (Huemer et al., 2020). Among these mechanisms, persister cell formation is a significant contributor to antibiotic resistance due to their ability to tolerate antibiotics and survive in hostile environments (Harms et al., 2016; Soares et al., 2020).

Persisters are a subset of microbial cells that possess the ability to survive lethal concentrations of antibiotics by transitioning into a slow or non-growing state through non-genetic and non-inheritable mechanisms (Wainwright et al., 2021). This phenotype is characterized through a biphasic killing curve, in which non-persistent are eliminated after exposure to lethal doses of bactericidal antibiotics, while persistent cells remain and resume growth after removal of the stressor, without any genetic variation (Paranjape and Shashidhar, 2019). The emergence of persister cells poses an important challenge in the field of bacterial infections, as these dormant cells show greater tolerance to antibiotics, leading to treatment failures and recurrent infections (Gollan et al., 2019; Khan et al., 2020).

*Salmonella* enterica serovar Typhimurium is a prevalent foodborne pathogen that induces gastroenteritis in humans and animals worldwide (Patra et al., 2021; Narimisa et al., 2024). This non-typhoidal *Salmonella* infection leads to morbidity and mortality in developed and developing nations alike (Santos et al., 2011). The treatment of this infection typically involves the use of a variety of antibacterial agents, including cephalosporins and fluoroquinolones (Pietsch et al., 2021; Toyting et al., 2023). However, the emergence of multi-drug resistant strains of *S*. Typhimurium is on the rise globally, primarily due to the excessive use of antibiotics (Patra et al., 2021; Wu and Hulme, 2021). Moreover, the presence of persister cells in this bacterium further complicates treatment outcomes and contributes to the persistence of infections (González et al., 2022).

The Lon protease, a highly conserved essential protease, belongs to ATP-dependent family of proteases and has been extensively studied in bacteria (Wlodawer et al., 2022). Lon is known to play crucial regulatory roles in bacterial cells by modulating levels and stability of regulatory proteins that govern gene expression and by maintaining cellular homeostasis through the degradation of misfolded proteins and regulation of protein turnover (Sauerbrei et al., 2020; Kirthika et al., 2023). Additionally, Lon protease has been implicated in the activation of type II toxin-antitoxin (TA) systems, which are pivotal for bacterial persistence and antibiotic tolerance (Równicki et al., 2020). The TA systems comprise a toxin that hinders crucial cellular processes and an antitoxin that neutralizes the toxin’s effects (Amraei et al., 2020; Amraei et al., 2023). When exposed to stressors like antibiotics, Lon protease can degrade antitoxin proteins, leading to the release of toxins and activation of the TA system. In the majority of instances, antitoxin genes are situated upstream of their corresponding toxin genes, with the transcription of TA operon being self-regulated through the binding of the antitoxin or the toxin-antitoxin complex to the promoter region. The alleviation of transcriptional repression occurs upon degradation of the antitoxin, leading to elevated production of both antitoxin and toxin transcripts. This activation via antitoxin degradation can result in various outcomes including growth inhibition, programmed cell death, and persistence (Ramisetty, 2020; Kamruzzaman et al., 2021).

By targeting the Lon protease, the activation of TA systems can be disrupted, consequently preventing the formation of persister cells and increasing bacterial susceptibility to antibiotics (Babin et al., 2019). Screening molecules for potential bioactivity is a costly and time-consuming process. Considering these challenges, a viable strategy for drug development involves repurposing of existing drugs or identifying novel indications for them (Rudrapal et al., 2020; Ng et al., 2021). Within this context, drug repositioning emerges as a promising approach to identify novel compounds capable of effectively targeting the Lon protease and disrupting persister cell formation in *S*. Typhimurium.

This study seeks to assess the efficacy of repurposed drugs in inhibiting Lon protease activity and decreasing persister cell formation in *S*. Typhimurium. Through the examination of TA system gene expression and phenotypic assays, we evaluated the potency of these repurposed drugs. Our findings offer potential for the development of new therapeutic strategies aimed at targeting persistent cells and preventing treatment failures in *Salmonella* infections.

## Materials and methods

### Bacterial strain and growth conditions

The bacterium used in this study was *S*. Typhimurium ATCC 14028. The strain was preserved in Brain Heart Infusion (BHI) broth (Merck, Darmstadt, Germany) supplemented with 20% glycerol at -80°C and cultured on Luria–Bertani (LB) agar plates at 37°C.

### Protein-ligand preparation and molecular docking analysis

The X-ray crystallography structures of the Lon protease (PDB code: 1RR9) were obtained from the RCSB Protein Data Bank at a resolution of 2.1 Å (Rose et al., 2016). The structures underwent preparation steps including hydrogen addition, removal of water molecules, and energy minimization using UCSF Chimera.

A ligand library was sourced from Drug Bank, consisting of 2588 drugs that were screened and their structures downloaded in SDF format. The ligands were optimized and converted to PDBQT format using the graphical user interface version of PyRx virtual screening tool.

### Molecular docking and virtual screening

Virtual screening docking is a method utilized to streamline the process and reduce costs associated with designing drugs against specific targets. PyRx software, with Autodock wizard as the docking engine, was used for molecular screening of all the ligands.

During docking, ligands were considered as flexible while the protein remained rigid. Grid parameters configuration files were generated using the Auto Grid engine in PyRx. The selected molecules were docked into the Lon protease’s active site based on literature reference (Botos et al., 2004). Default docking settings were applied to create 10 docked conformations per chemical. The ligand with the highest binding energy (most negative) was considered to have the maximum binding affinity. At the end of docking, we considered several factors such as binding energy, and hydrogen bonds. Following an in-depth examination of ligand-receptor interactions, the ligand-receptor complexes were saved for visualization using UCSF Chimera.

### Molecular dynamics simulation

In this research, molecular dynamics simulations were conducted on both the unbound and specific docked complexes utilizing GROMACS software with the CHARMM27 force field. The protein and protein-ligand complexes were immersed in a cubic box filled with TIP3P water molecules. To maintain a physiologically relevant ionic strength of 0.15 M and neutralize the system’s charge, appropriate quantities of Na+ and Cl- ions were included.

The energy minimization phase consisted of 50,000 steps employing the steepest descent method for each simulation (Meza, 2010). The simulations were carried out at a temperature of 300 K, following equilibration in two stages: NVT and NPT at 300 K for 100 ps. Throughout the simulations, the Berendsen thermostat (Berendsen et al., 1987) and Parrinello-Rahman barostat (Parrinello and Rahman, 1981) were utilized to regulate pressure and temperature. Subsequently, 100 ns simulations were executed with a time step of 2 femtoseconds (fs). The Lincs algorithm (Hess et al., 1997) was employed to compute van der Waals interactions, long-range electrostatics, and covalent bond constraints. Finally, the resulting molecular dynamics simulation trajectories were analyzed using GROMACS utilities to extract relevant insights from the data.

### Determination of minimum inhibitory concentration

Antibiotic powders, including Ceftazidime, Ciprofloxacin, Colistin, Nafcillin, and Diosmin, were acquired from Sigma-Aldrich (Taufkirchen, Germany). The minimum inhibitory concentrations (MICs) of these antibiotics against *S*. Typhimurium were assessed using the broth microdilution method in 96 U-shaped well plates. To determine the MIC, 100 μL of Mueller-Hinton Broth (MHB) (Merck Co., USA) was dispensed into each well of a 96-well plate. Subsequently, 100 μL of each antibiotic solution was added to the first well for serial dilution. Next, 100 μL of an overnight culture containing approximately 10^5 cells were introduced into each well, and the plates were incubated at 37°C for 24 hours. The MIC was identified as the lowest antibiotic concentration without visible growth. Each antibiotic’s MIC value was determined independently three times.

### Persister formation assays

Persisters, a subpopulation of bacteria with enhanced tolerance to antibiotics, were identified through time-dependent killing experiments. A single colony of *S*. Typhimurium was cultured in 5 ml of LB broth for 24 hours. The overnight culture was then diluted 1:100 in fresh LB broth and incubated at 37°C on a shaker at 200 rpm until reaching an optical density of 0.25 at 600 nm during the logarithmic growth phase. Subsequently, these cultures were treated separately with 100 µg/ml ceftazidime (50 X MIC), 50 µg/ml colistin (50 X MIC), and 6.25 µg/ml ciprofloxacin (50 X MIC). Furthermore, to determine the impact of Nafcillin and Diosmin on the viability of *S*. Typhimurium, the bacterial culture was exposed to varying concentrations of these compounds separately. In addition, to assess the effect of combining Nafcillin or Diosmin with the studied antibiotics on persister cell formation in *S*. Typhimurium, cultures in the exponential growth phase were exposed to 50-fold MIC concentrations of Ceftazidime, Ciprofloxacin, and Colistin in the presence of Nafcillin (64 µg/ml) and Diosmin (500 µg/ml) individually.

After treatment, the cultures were washed three times with 0.85% sterile saline solution. Viable cell counts were determined at different time points (1, 2, 3, 5, and 24 hours) by serial dilution in Phosphate-Buffered Saline (PBS) and plating onto LB agar for colony enumeration. A bacterial culture without any treatments served as the control group.

### Relative qRT-PCR analysis

The relative expression levels of the TA systems and Lon protease of *S*. Typhimurium were evaluated through qRT-PCR. The primer sequences utilized in this study can be found in **Table 1**. Total RNA was isolated from 1 mL of *S*. Typhimurium during the exponential phase after a 3-hour exposure to Ceftazidime (50X MIC), Colistin (50X MIC), Ciprofloxacin (50X MIC), Nafcillin (64 µg/mL), and Diosmin (500 µg/mL). Additionally, total RNA was extracted from *S*. Typhimurium cultures subjected to a combination of any of the antibiotics mentioned above with Nafcillin or Diosmin. Untreated *S*. Typhimurium served as the control.

**Table 1:** Primers used for qRT-PCR.

Primer Name	Primer Sequence	Product size (bp)	Reference
*gnat*	F: TGTTATCTGCCGGAAAGCGT R: TGGTACTGCGCCAAATCCTT	119	(Narimisa et al., 2021a)
*rhh*	F: AGCAAACCAACCTGACGGAT R: GCGCTCCGTCAGATATACCC	95	(Narimisa et al., 2021a)
*vapC*	F: CGAAATCATTGCGGTTGGCA R: TCCTGTACTGCGGGTTGTTC	126	(Narimisa et al., 2021a)
*vapB*	F: AACACCTGTCGGGCCTTATG R: ATGCGTTCAAATTCGCGGAG	96	(Narimisa et al., 2021a)
*relE*	F: CGCTTCTGATTCACCACCCT R: TCGCAGGCATTCATCCGTTA	140	(Narimisa et al., 2021a)
*parE*	F: ATGTCCTGGCTGAAATGGGG R: ATCAACGCCAGCTTCGCTAT	149	(Narimisa et al., 2021a)
*invA*	F: AGCGTACTGGAAAGGGAAAG R: ATACCGCCAATAAAGTTCACAAAG	116	(Kasturi and Drgon, 2017)
*phd*	F: TGGTAACAAACGTACCGCCT R: GGATTCGCAGCGAGTGAGAT	75	This study
*doc*	F: GAAGTCCTGGAAAGTGCGGT R: GCTGATGATCACTGCGGACT	78	This study
*relE*	F: GAACGGCTTGAAAACCCTCG R: CGCTGTACACCAGCCTGTAT	103	This study
*relB*	F: TGGGAACTTTCAATGCCGGA R: TGAGCATAAAGTGCCGGAGG	94	This study
*hha*	F: TTACGGCGCTGTCAGACAAT R: TTCTGCAAGACGGTGATCCG	117	This study
*tomB*	F: CGACCTCAGCAGTCAACCTG R: GTTGCCCGATTTACGCCATT	197	This study
*lon protease*	F: AATAACCTCAACCAGCGCGA R: TAGGTCAGGCAAACACGGAC	116	This study

Total RNA from *S*. Typhimurium cultures was extracted using the TRIzol reagent method (Rio et al., 2010). Subsequently, DNase1 treatment was administered (Thermo Scientific, USA) following the protocol. The RNA was then reverse-transcribed using the AddScript cDNA synthesis kit (AddBio; South Korea) as per the manufacturer’s instructions.

Quantitative reverse transcription-PCR (qRT-PCR) was conducted with three technical replicates for each sample using a Rotor-Gene thermal cycler (Corbett Life Sciences, Sydney, Australia) with the SYBR Green method (Ampliqon Co, Denmark). The total reaction volume (20 μL) included 1 μL of cDNA, 10 μL of SYBR Green master mix, 7 μL of nuclease-free water, and 1 μL of each primer. The thermal cycling program comprised an initial denaturation at 95°C for 12 minutes, followed by 40 cycles at 95°C for 10 seconds, 60°C for 20 seconds, and 72°C for 25 seconds. The *invA* gene was utilized as the reference gene for normalizing expression levels. The relative fold changes in expression levels were determined using the delta-delta Ct method (Rao et al., 2013).

### Statistical analysis

Data analysis and statistical chart plotting were performed using GraphPad Prism 8 (GraphPad Software, Inc). One-way analysis of variance (ANOVA) followed by Tukey’s *post-hoc* test for multiple comparisons was employed to assess differences in gene expression levels and the final colony count after 24 hours. Results were presented as mean ± standard deviation (SD).

## Results

### The best docking scores of diosmin and nafcillin with lon protease of *S*. Typhimurium

**Figure 1:**
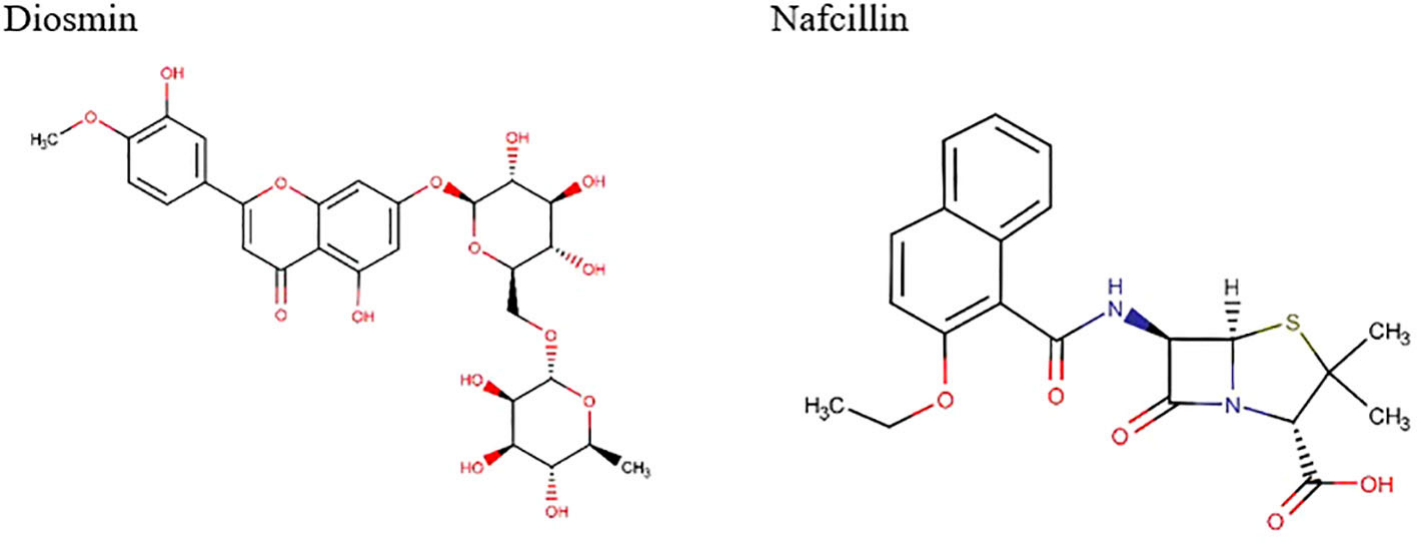
Chemical structures of Diosmin and Nafcillin.

The docking study conducted with FDA-approved drugs as ligands against the Lon protease of *S*. Typhimurium provided valuable insights into molecular interactions. PyRx docking was performed with a grid box formation to encompass the active site residues of the protein as described in the study by Botos et al (Botos et al., 2004). The interaction studies primarily focused on assessing the effective binding of our chosen ligands with the binding site residues within the grid box, thereby evaluating a suitable and effective docking methodology. A total of 2588 drugs from Drug Bank were initially acquired. Following the elimination of redundant drugs, 44 unique drugs with a binding affinity equal to or greater than -6.5 kcal/mol for Lon protease were identified. **Table 2** displays the names and binding affinities of the top seven ligands for Lon protease. However, several drugs among the top hits did not interact with the amino acids crucial to the enzyme’s active site, such as amino acids 722 and 679 according to Botos et al. Findings indicated that two drugs, Diosmin and Nafcillin (**Figure 1**), formed hydrogen bonds with the mentioned amino acids. Specifically, Diosmin engaged in hydrogen bonding with active site residues of Lon protease including Lys 722 and Ala 679, while Nafcillin interacted with residue Lys 722 through hydrogen bonding, positioning them as promising ligands for further exploration.

**Table 2:** docking scores of top 7 drugs after virtual screening.

No	DrugBank ID	Name	Binding affinity (KCal/mol)	Key residues interraction
1	DB08995	Diosmin	-7.9	Lys 722 and Ala 679
2	DB00607	Nafcillin	-7.7	Lys 722
3	DB13345	Dihydroergocristine	-7.4	No H-bond interaction
4	DB12161	Deutetrabenazine	-7.2	No H-bond interaction
5	DB08834	Tauroursodeoxycholic acid	-6.9	No H-bond interaction
6	DB04570	Latamoxef	-6.7	No H-bond interaction
7	DB16691	Nirmatrelvir	-6.7	No H-bond interaction

**Figure 2:**
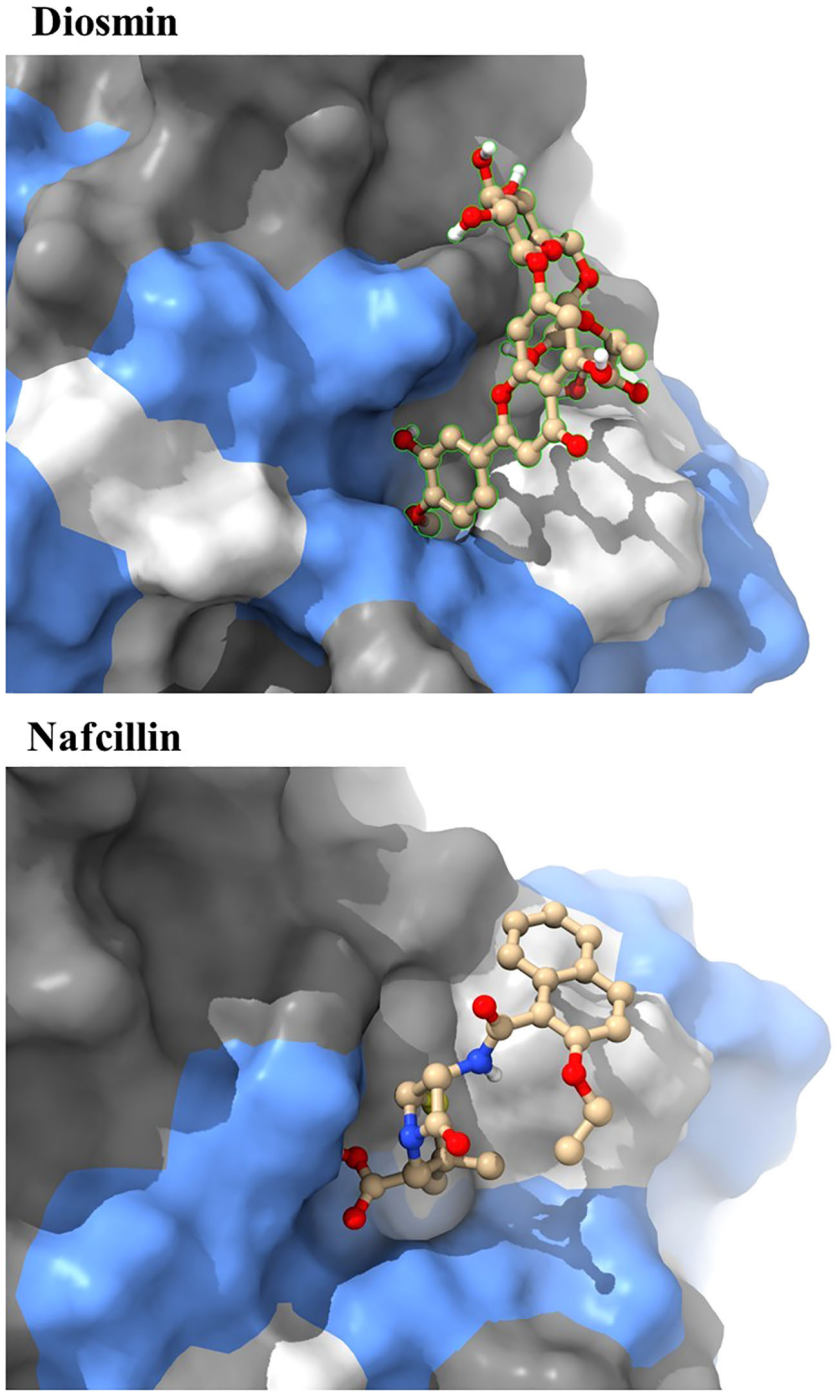
The best binding pose of molecular docking result of Diosmin and Nafcillin within binding pocket of the Lon protease, indicating potential interactions and binding orientations of the compounds within the active site of the enzyme.

**Figure 3:**
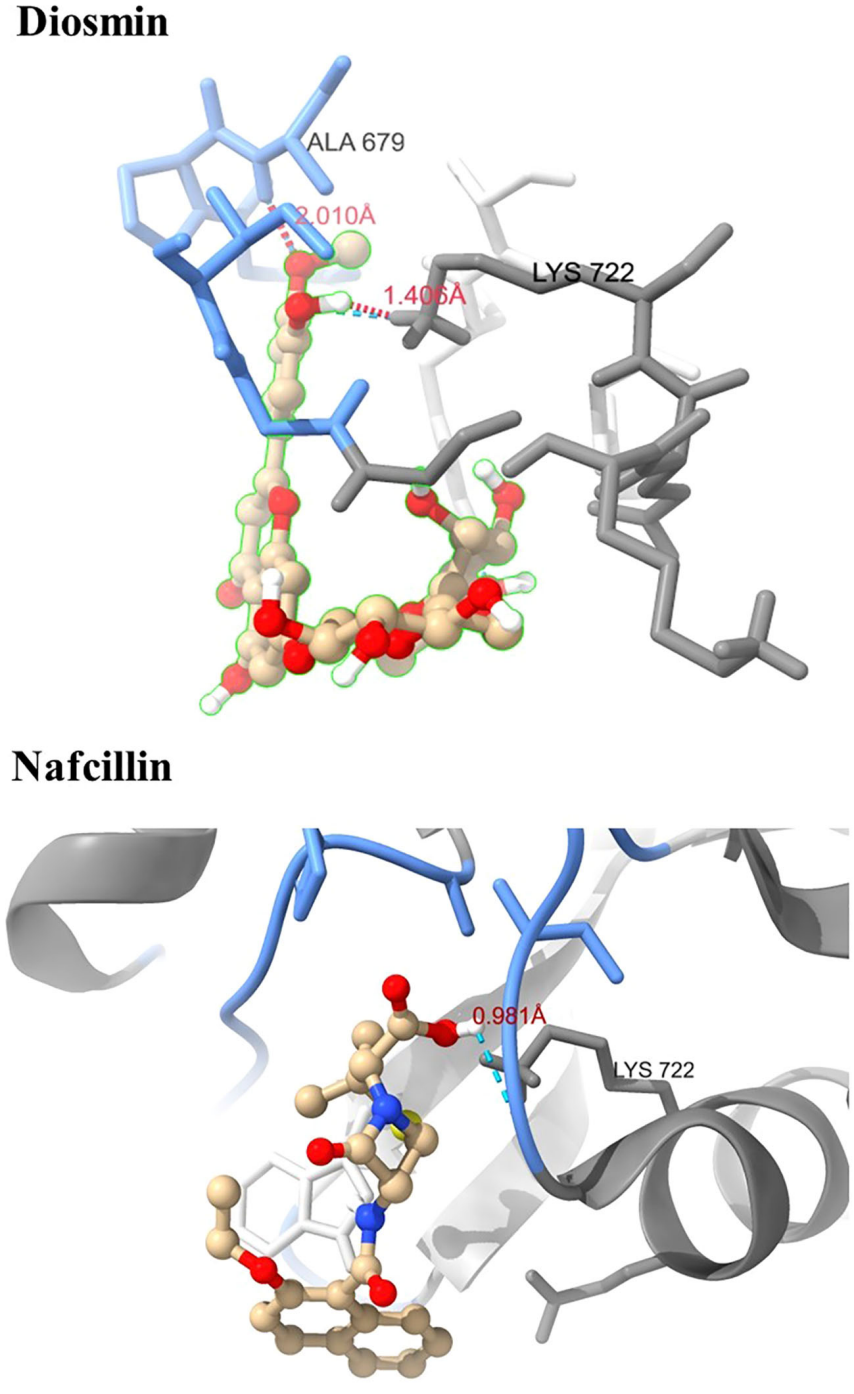
Three-dimensional interactions of Diosmin and Nafcillin within the binding pocket of the Lon protease. Hydrogen-bonded interactions are shown as dotted lines. Diosmin forms hydrogen bonds with Lys 722 and Ala 679, while Nafcillin interacts with Lys 722 through hydrogen bonding.

**Figure 4:**
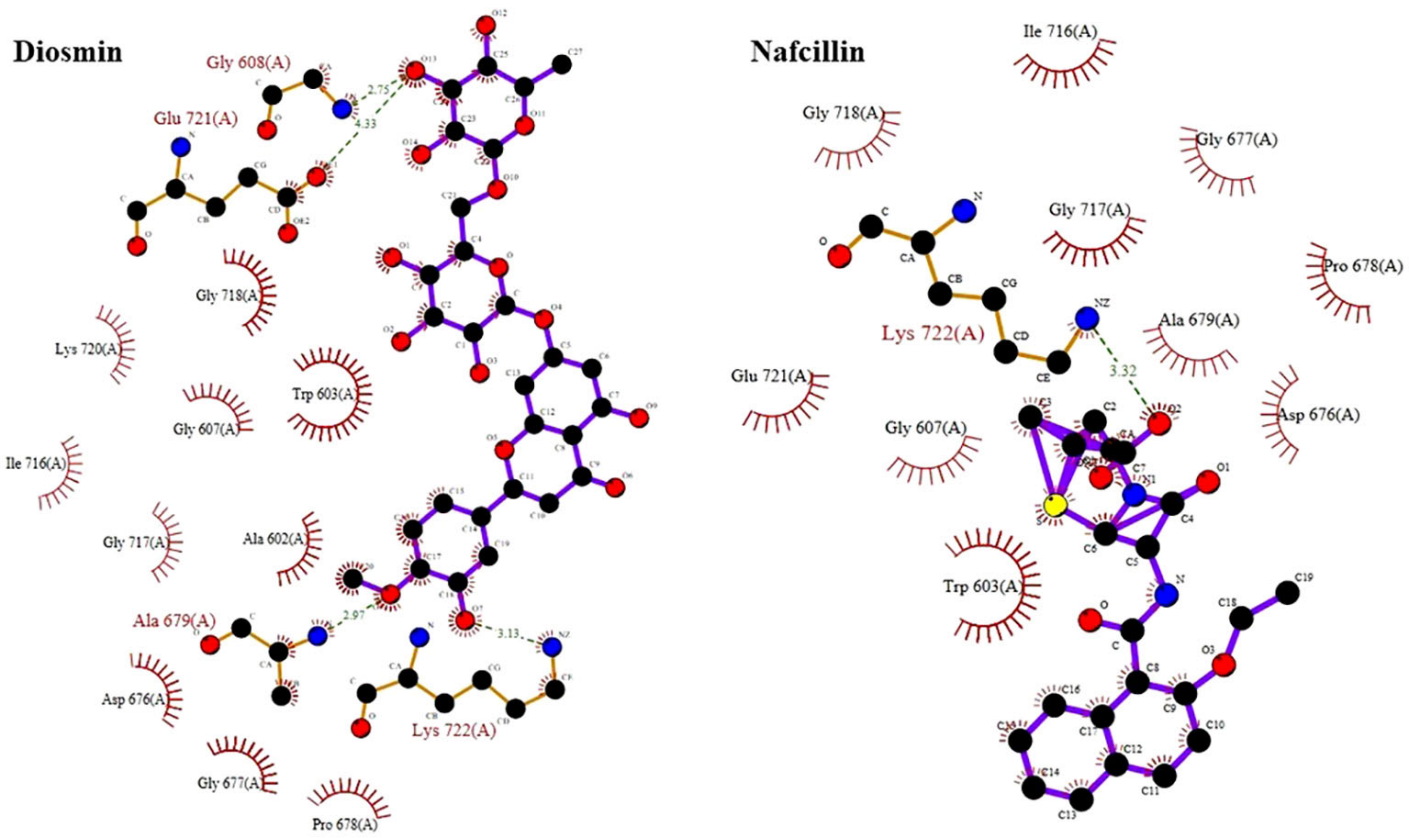
Detailed view of the interactions of Diosmin and Nafcillin with Lon protease generated by ligplot. Ligand is shown in purple and: green dashed lines symbolize hydrogen bonds distances measured in angstroms (Å), red spoked arcs represent hydrophobic contacts; and atoms are color-coded as black for carbon, blue for nitrogen, red for oxygen, and yellow for sulfur.

Visual rescoring using UCSF Chimera was conducted to confirm the binding of the selected drugs with the active site residues of their respective proteins. The docking patterns and hydrogen bonding interactions of Diosmin and Nafcillin are depicted in **Figures 2** and **3**, respectively. Additionally, Ligplot analysis illustrated the formation of hydrogen bonds between the selected ligands and their target protein (**Figure 4**).

### Stability of lon-nafcillin and lon-diosmin complexes during molecular dynamics simulation

**Figure 5:**
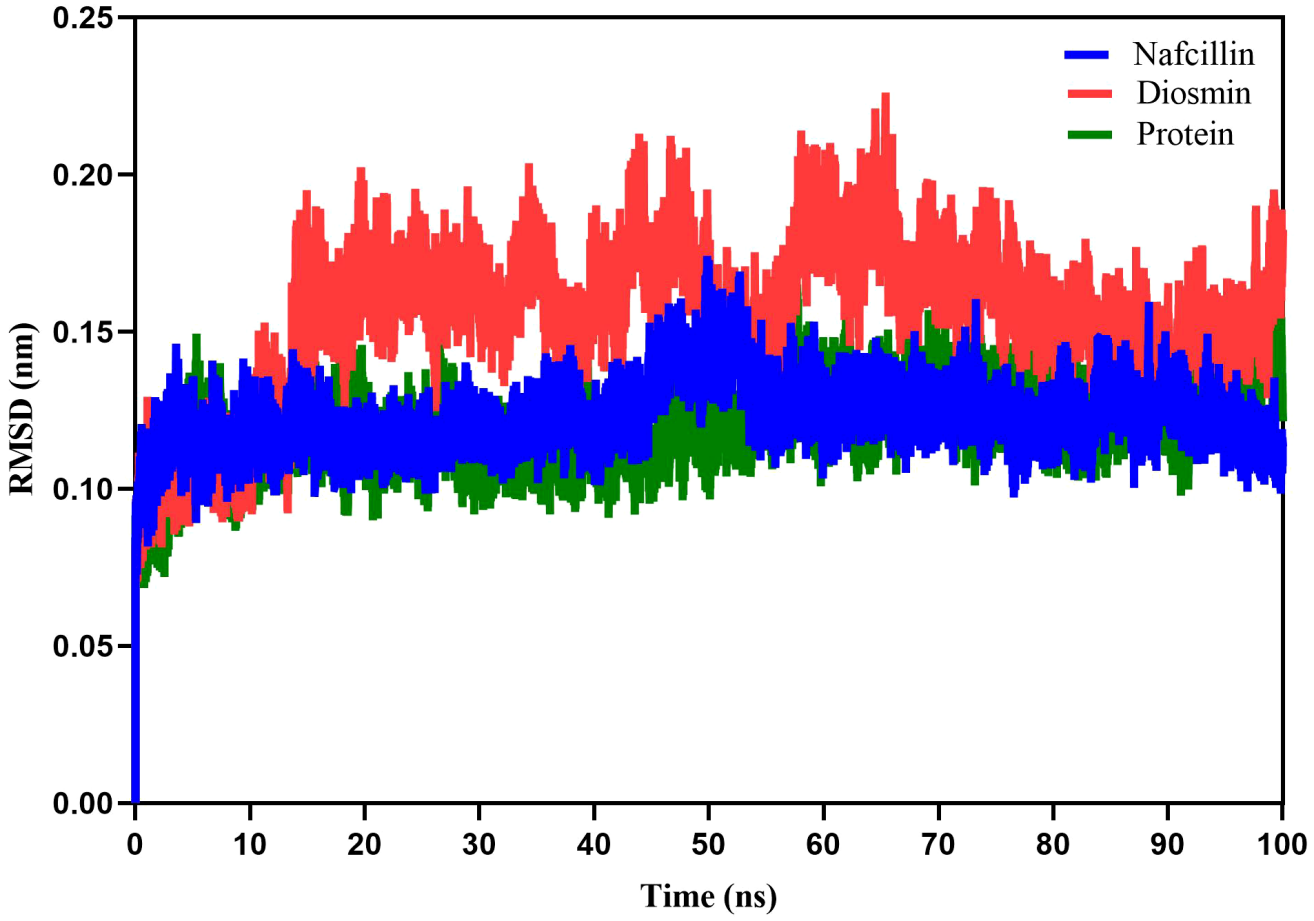
Root-mean-square deviation (RMSD) plots showing the backbone atoms of the Lon protease in its free form (green), and in the presence of Nafcillin (blue) and Diosmin (red) from 100 ns molecular dynamics simulations. The binding of Diosmin and Nafcillin did not induce instability in the enzyme’s structure.

Following the docking analysis, the most effective ligands against the Lon protease were selected for further molecular dynamics (MD) simulation studies. **Figure 5** illustrates the root mean square deviation (RMSD) plots of the Lon protease in its free form and in the presence of Diosmin and Nafcillin during a 100 ns MD simulation. The RMSD analysis provided insights into the structural changes of the protein, confirming its stability and equilibrium during the simulation (Khan et al., 2021).

The average RMSD values for the unbound Lon protease, Lon-Nafcillin, and Lon-Diosmin were 0.120, 0.122, and 0.154, respectively. Analysis of the results indicated a slight increase in the mean RMSD for Lon protease in the presence of Diosmin, while Nafcillin binding showed minimal impact on the mean RMSD. Overall, it can be concluded that the binding of Diosmin and Nafcillin did not induce instability in the enzyme’s structure, as evidenced by the stable RMSD values observed during the MD simulations with the ligands.

**Figure 6:**
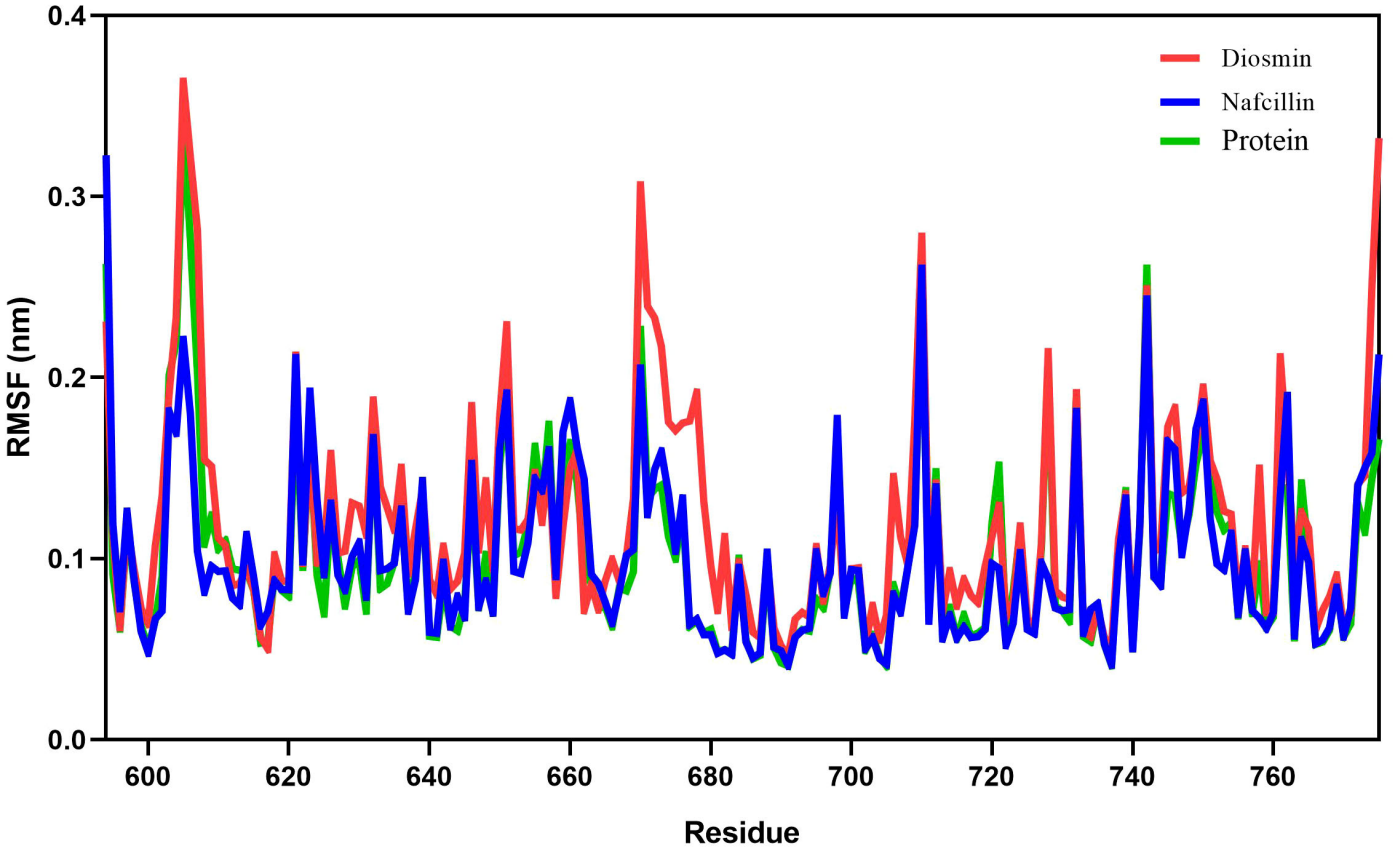
Root-mean-square fluctuation (RMSF) plots showing the fluctuations of the backbone atoms of the Lon protease in its free form (green), and in the presence of Nafcillin (blue) and Diosmin (red) from 100 ns molecular dynamics simulations. Consistent residues were identified during the interaction of Lon protease with Diosmin and Nafcillin.

Root mean square fluctuation (RMSF) analysis was employed to evaluate the fluctuation of protein residues and the dynamic stability of the systems throughout the 100 ns simulation process (Priya et al., 2015). The RMSF values for Lon protease in its free form and in the presence of Diosmin and Nafcillin are depicted in **Figure 6**. The RMSF values for Lon protease, Lon-Nafcillin, and Lon-Diosmin were 0.10, 0.10, and 0.12, respectively. These values indicated that the complexes Lon-Nafcillin and Lon-Diosmin remained stable throughout the simulation period.

**Figure 7:**
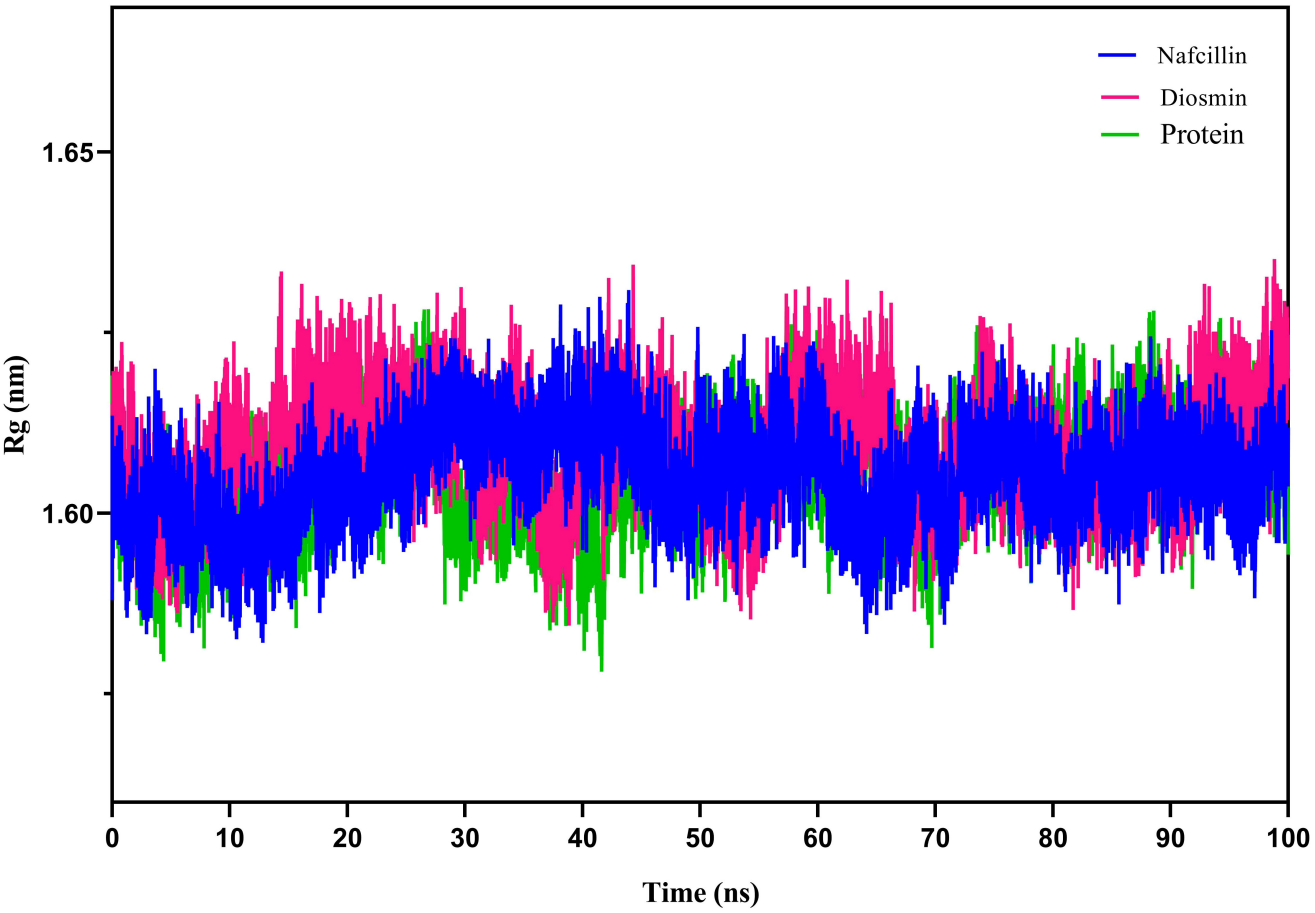
Radius of gyration (Rg) plots showing the backbone atom compaction of the Lon protease in its free form (green), and in the presence of Nafcillin (blue) and Diosmin (red) from 100 ns molecular dynamics simulations. The structure of the enzyme remains unchanged in terms of compaction upon interaction with the ligands.

To assess the compactness of the protein during the simulation period, the radius of gyration (Rg) of Lon protease in its free form and in the presence of Nafcillin and Diosmin was calculated (**Figure 7**). The Rg values for Lon protease, Lon-Nafcillin, and Lon-Diosmin were 1.605, 1.609, and 1.605, respectively. The Rg values remained within narrow ranges for Lon protease in complex with the compounds, showing no significant upward or downward trend during the simulation time.

**Figure 8:**
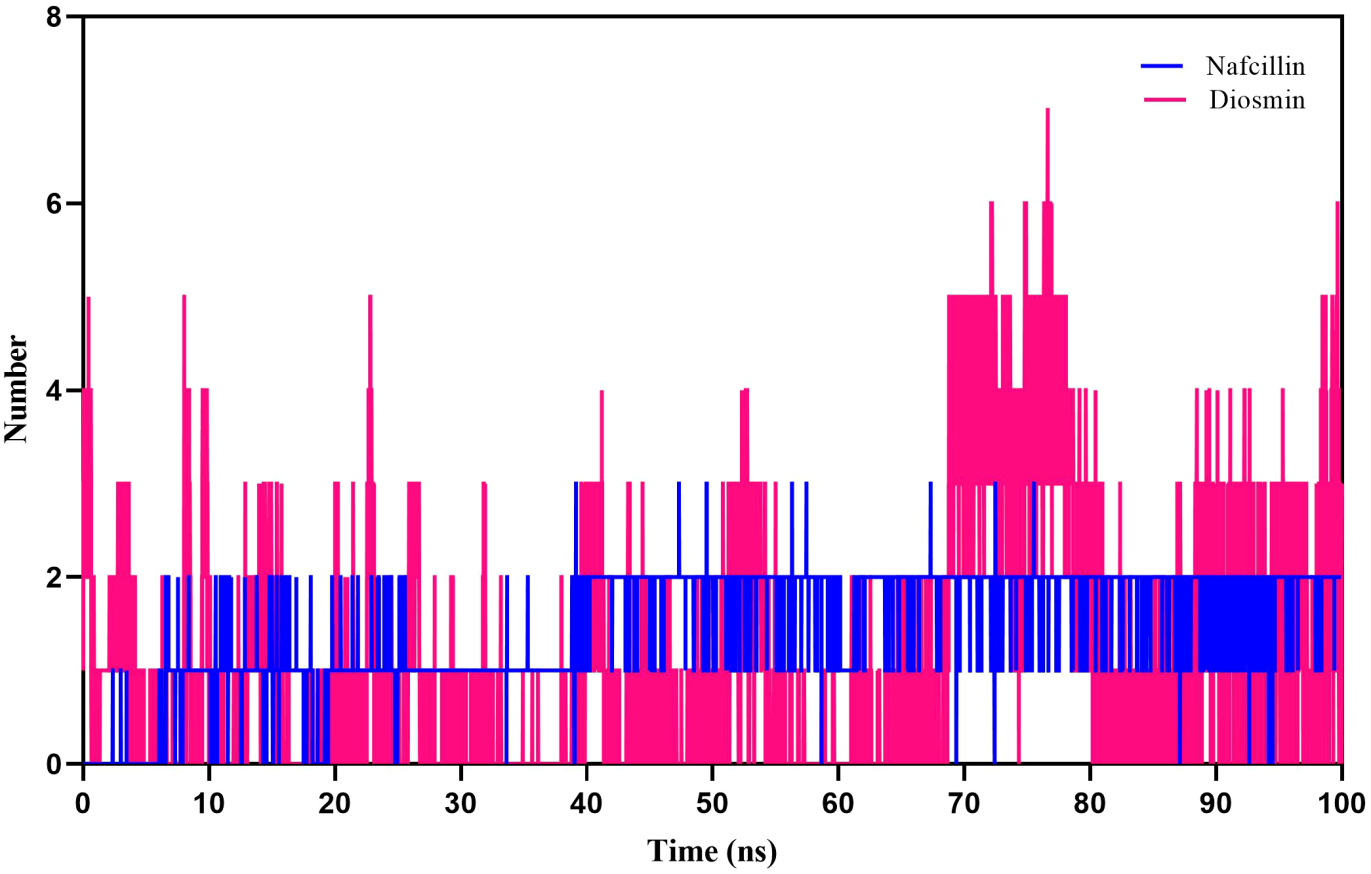
Number of hydrogen bonds formed between Lon protease atoms and Nafcillin (blue) and Diosmin (red) from 100 ns molecular dynamics simulations. Diosmin forms the most stable structure.

Furthermore, hydrogen bond formation plays a crucial role in stabilizing protein-ligand complex structures by minimizing system energy (Chen et al., 2016). The hydrogen bond interactions of the complexes were analyzed to validate the ligands’ affinity to inhibit the proteins. The hydrogen bond patterns between bound Lon protease with Nafcillin and Diosmin were studied, and **Figures 8** depict the number of hydrogen bonds versus time during the simulation. The stability of hydrogen bonds in both complexes indicates that the binding of these compounds to Lon protease remained stable throughout the simulation time. Interestingly, a higher number of hydrogen bonds were formed between Diosmin and the enzyme compared to Lon-Nafcillin, suggesting that Diosmin may have a more potent inhibitory effect on Lon protease than Nafcillin.

**Figure 9:**
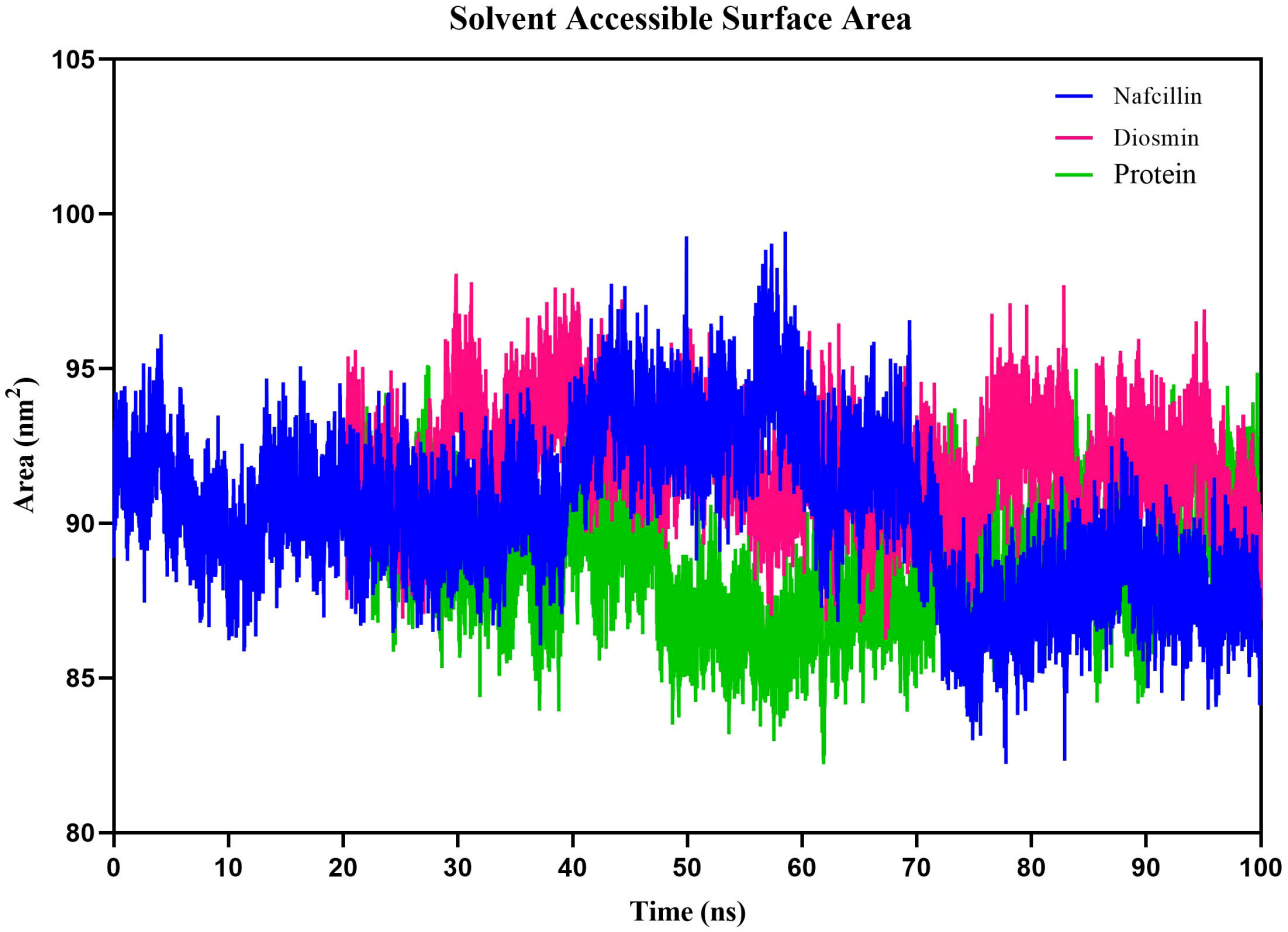
Solvent Accessible Surface Area (SASA) plots of the backbone atoms of Lon protease in its free form (green) and in the presence of Nafcillin (blue) and Diosmin (red) from 100 ns molecular dynamics simulations. The SASA values indicate that the binding of Nafcillin to Lon protease leads to an increase in the exposed surface area compared to the unbound state.

Additionally, we examined the solvent-accessible surface area (SASA) of protein-ligand complexes to evaluate the contribution of hydrophobic interactions of nonpolar amino acids to protein conformation stability in a solvent environment (Ausaf Ali et al., 2014). The SASA results for Lon protease, Lon-Nafcillin, and Lon-Diosmin were 90.38, 92.10, and 89.23 nm2, respectively (**Figure 9**). The SASA values suggest that the binding of Nafcillin to Lon protease leads to an increase in the exposed surface area compared to the unbound state, as indicated by the SASA value of 92.10 nm2. On the other hand, the binding of Diosmin leads to a decrease in the exposed surface area, as indicated by the SASA value of 89.23 nm2. These results suggest that Nafcillin may cause conformational changes or induce a more open conformation in Lon protease, while binding Diosmin may cause a more stability of the complex.

### Reduction of the number of persister cells in *S*. Typhimurium in the presence of diosmin and nafcillin

The minimum inhibitory concentrations (MICs) of Ceftazidime, Ciprofloxacin, Colistin, and Nafcillin against *S*. Typhimurium ATCC 14028 were determined using a broth microdilution method. The MIC values for Ceftazidime, Ciprofloxacin, Colistin, and Nafcillin were 2 μg/ml, 0.125 μg/ml, 1 μg/ml, and 16 μg/ml, respectively. Notably, bacterial growth was observed at all tested concentrations of Diosmin.

To assess the viability of *S*. Typhimurium in the presence of Nafcillin and Diosmin, time-dependent killing experiments were conducted by exposing log-phase *S*. Typhimurium to three concentrations of Nafcillin (32, 64, and 128 μg/ml) and three concentrations of Diosmin (100, 250, and 500 μg/ml) over a 24-hour period.

**Figure 10:**
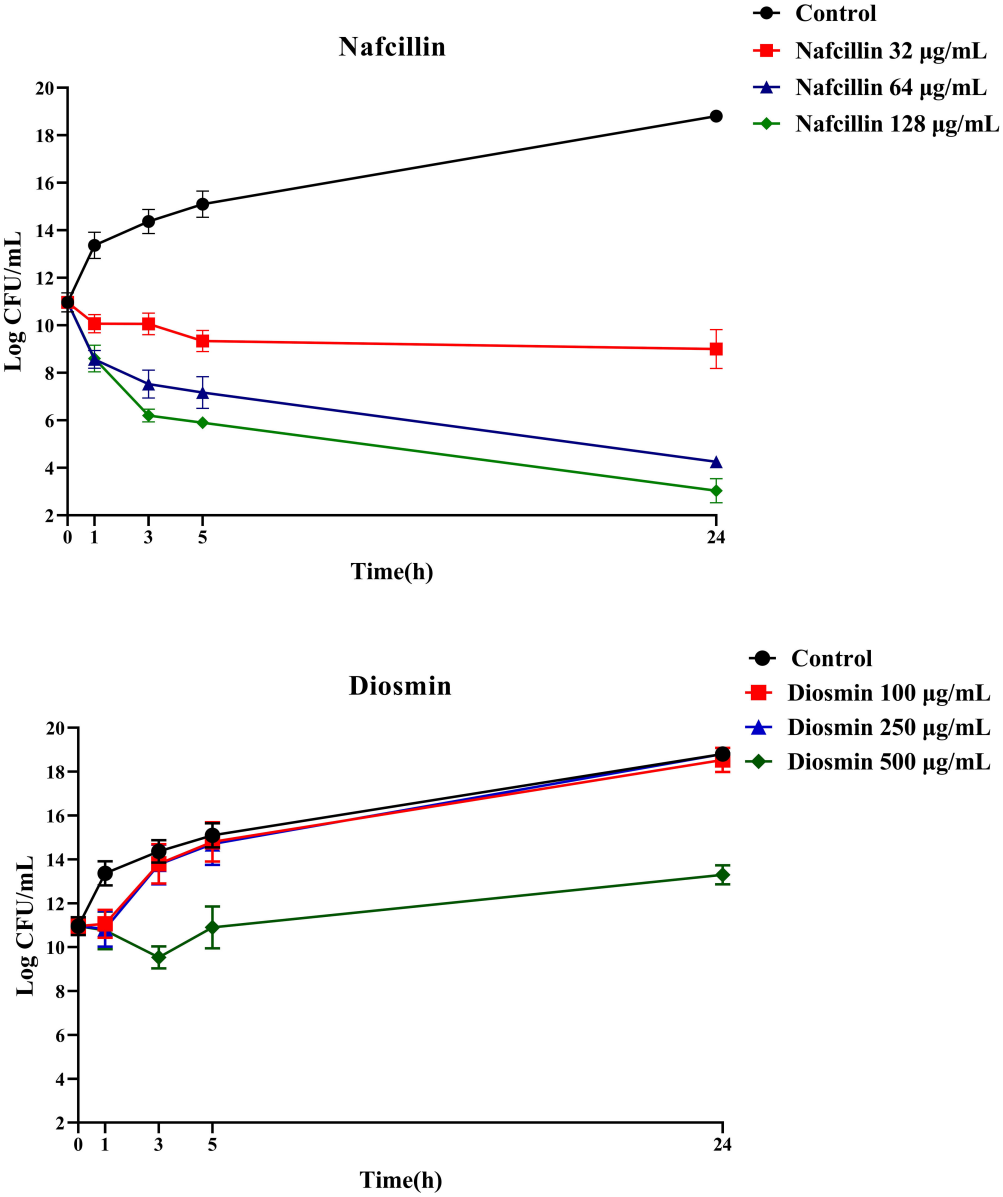
Time-dependent killing of *S*. Typhimurium when exposed to varying concentrations of Nafcillin and Diosmin. The bacterial culture without any antibiotic was used as a control. The reported values represent the averages from three independent biological replicates, with error bars denoting the standard deviation.

**Figure 10** illustrates that at 24 hours, Nafcillin concentrations of 64 and 128 μg/ml significantly reduced the number of viable cells. Specifically, a 15.7 log decrease was observed at 128 μg/ml compared to the control, a 14.6 log decrease at 64 μg/ml compared to the control, and a similar decrease at 32 μg/ml. In contrast, at concentrations of 100 μg/ml and 250 μg/ml of Diosmin, bacterial growth was comparable to the control; however, at 500 μg/ml, a reduction in living cells was evident with a 5.5 log decrease after 24 hours.

**Figure 11:**
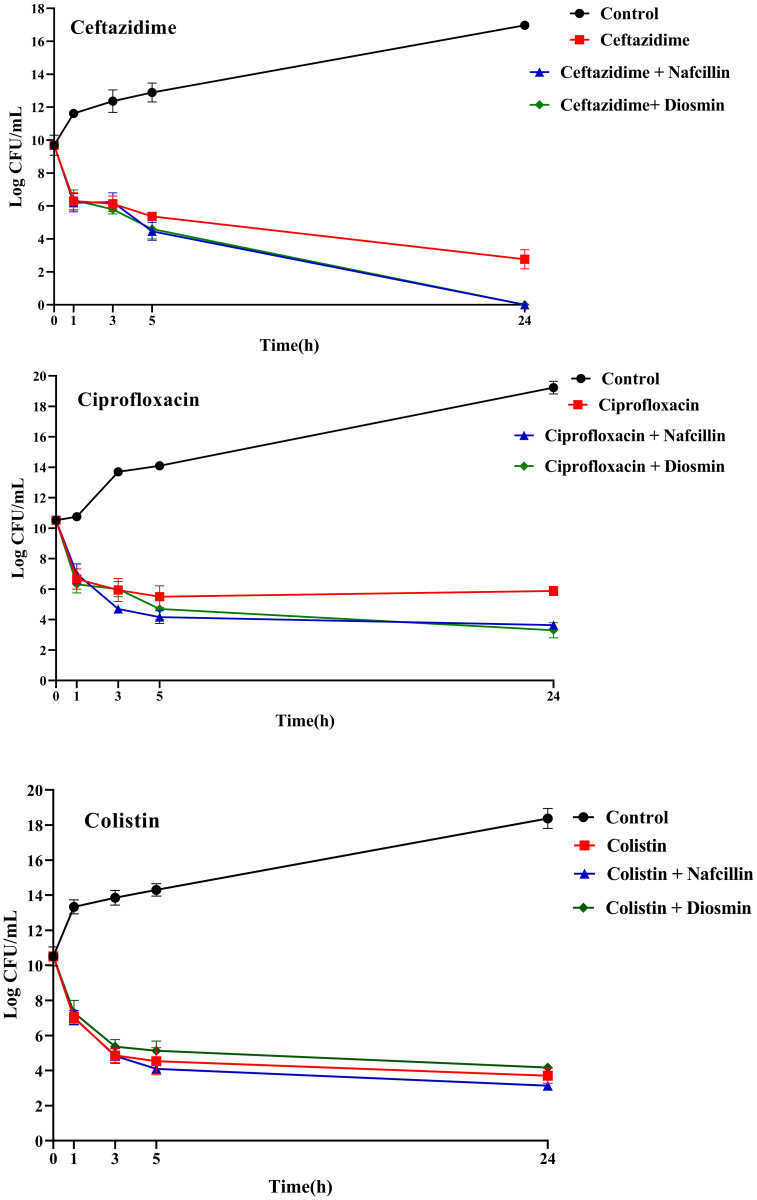
Time-dependent killing of *S*. Typhimurium exposed to different antibiotics and antibiotics with Nafcillin and Diosmin. The bacterial culture without any antibiotic was used as a control. The reported values represent the averages from three independent biological replicates, with error bars denoting the standard deviation.

Furthermore, the impact of combining Nafcillin (64 μg/ml) and Diosmin (500 μg/ml) with various antibiotics on persister cell formation was investigated. The results demonstrated that the combinations of Ceftazidime with Nafcillin or Diosmin significantly reduced bacterial cell numbers (P<0.0001), leading to the absence of viable cells after 24 hours. Similarly, the combination of Ciprofloxacin with Nafcillin and Diosmin significantly decreased bacterial cell counts (P<0.0001), resulting in a 2.5 log reduction in persister cells compared to antibiotic treatment alone. However, combinations involving Nafcillin (P=0.35) or Diosmin (P=0.50) with Colistin did not significantly reduce live bacterial cells after 24 hours, as shown in **Figure 11**.

### Suppression of type II TA systems gene expression in the presence of diosmin and nafcillin

**Figure 12:**
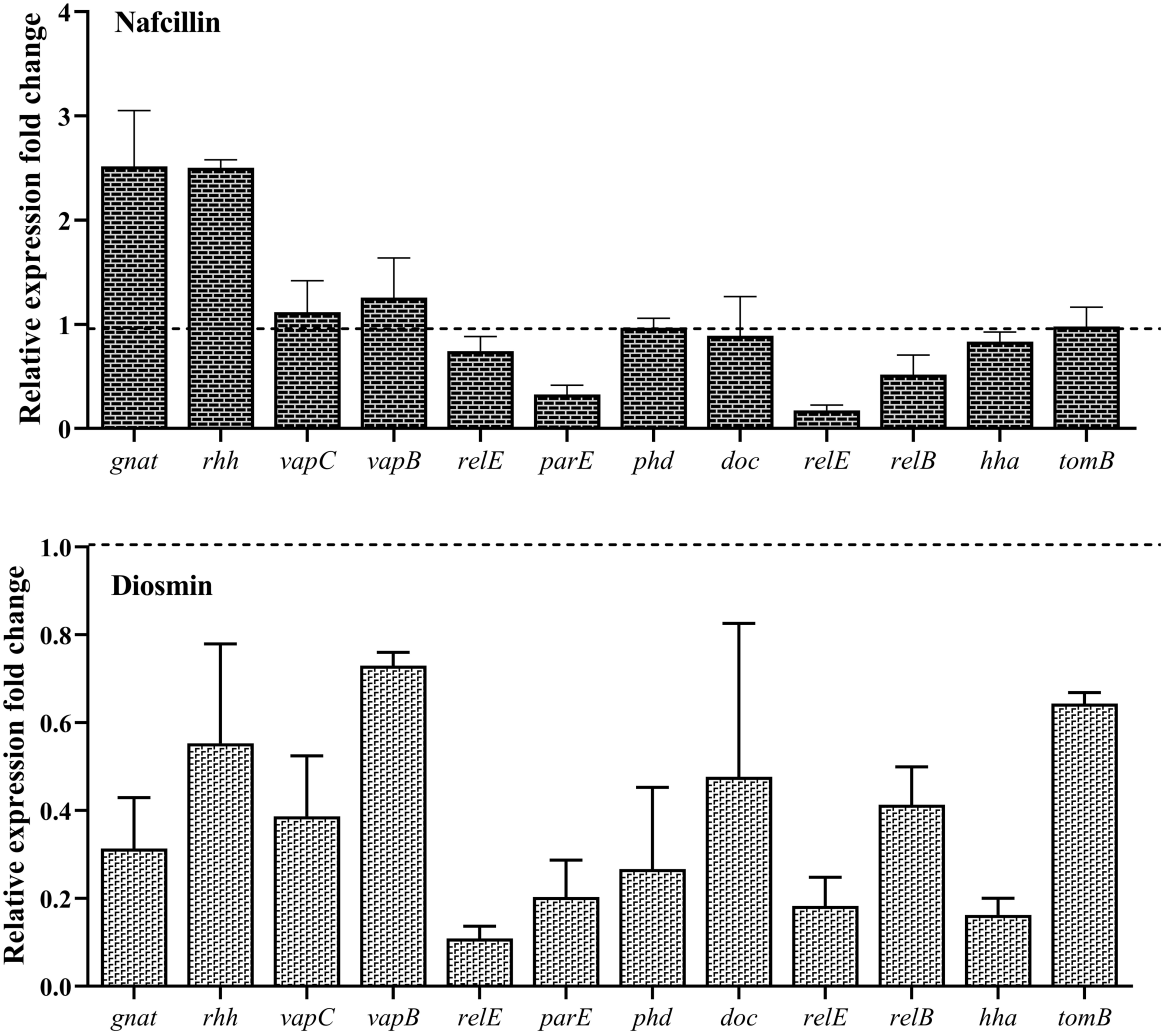
Analysis of the relative expression levels of type II TA system genes in the presence of Nafcillin and Diosmin. The relative expression is normalized using the reference gene invA. Error bars represent the standard deviations of three biological replicates. (*P*<0.0001 for Nafcillin and *P*=0.0002 for Diosmin using one-way ANOVA).

To assess the impact of Nafcillin and Diosmin on the expression of type II TA system genes, *S*. Typhimurium ATCC 14028 was exposed to Nafcillin (64 μg/ml) and Diosmin (500 μg/ml) for three hours. Our findings revealed a decrease in the expression levels of genes encoding type II TA systems in the presence of Diosmin. Specifically, exposure to Nafcillin led to a slight increase in the expression level of *gnat/rhh*, while reducing the expression levels of other studied type II TA system genes (**Figure 12**).

**Figure 13:**
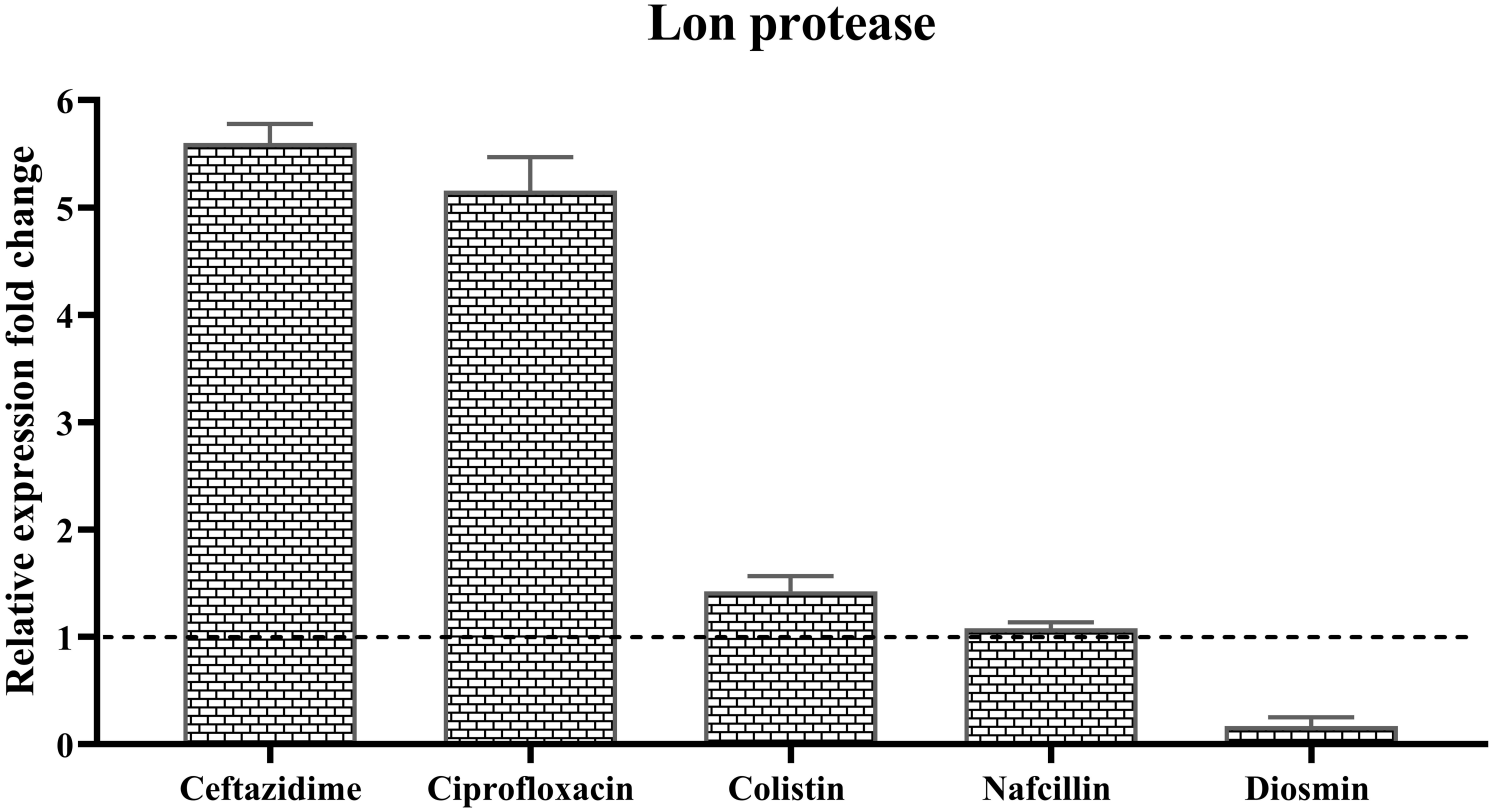
Analysis of the relative expression levels of of *lon protease* in the presence of different antibiotics. The relative expression is normalized using the reference gene invA. Error bars represent the standard deviations of three biological replicates. (*P*<0.0001 using one-way ANOVA).

Furthermore, the analysis of the relative expression level of *lon protease* in the presence of different antibiotics demonstrated varying effects. The highest increase in expression was observed in the presence of Ceftazidime with a 5.6-fold upregulation, followed by a 5.1-fold increase with Ciprofloxacin. Colistin resulted in a slight 1.4-fold increase in Lon protease expression, while Nafcillin showed minimal change in *lon* expression, and Diosmin led to a reduction in the expression level of this protease (**Figure 13**).

**Figure 14:**
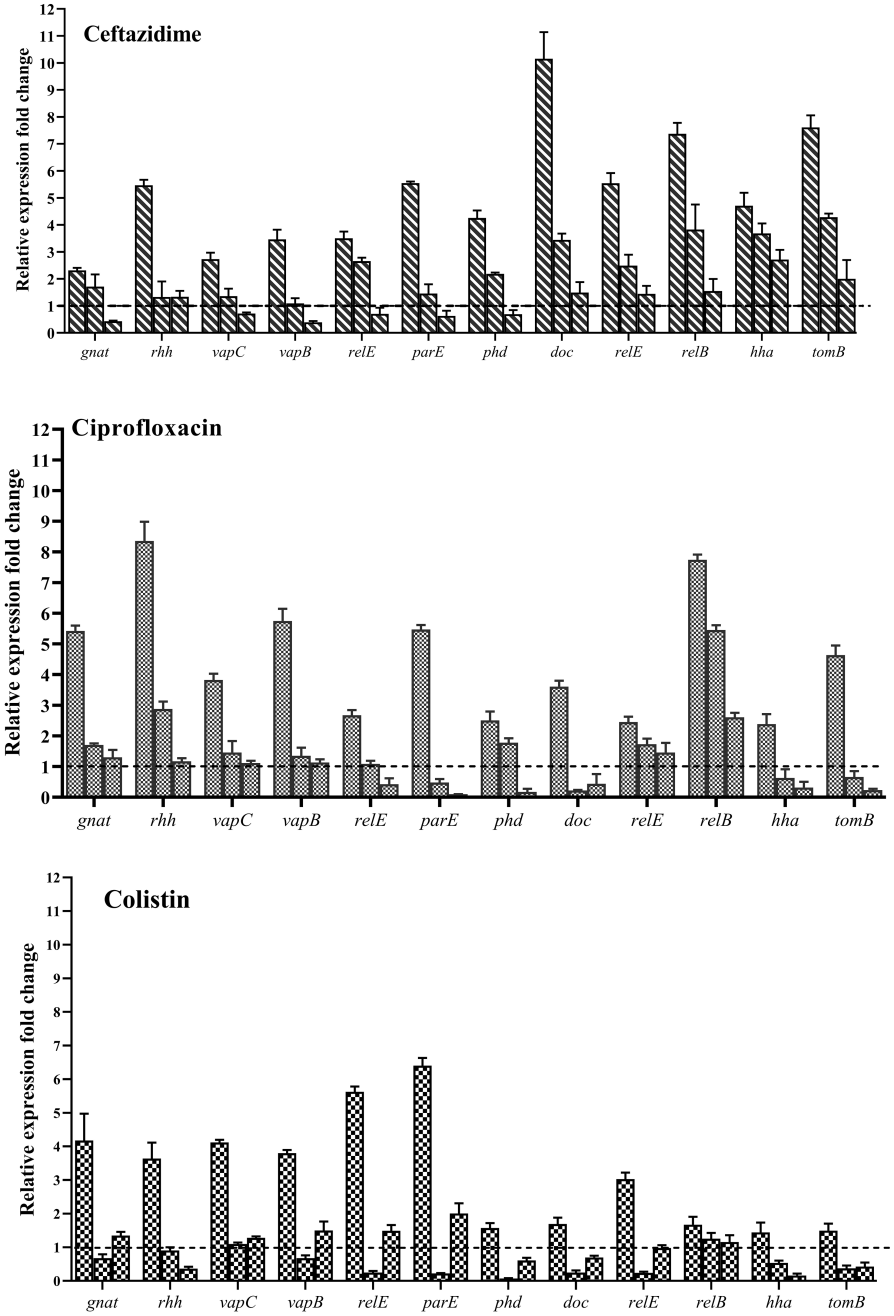
Analysis of relative expression level of type II TA system genes in the presence of various antibiotics. Each group of genes is represented by three bars: the first bar representing the antibiotic alone, the second bar representing the combination of the antibiotic with Nafcillin, and the third bar representing the combination of antibiotic with Diosmin. Normalization of relative expression was performed using the reference gene invA, and error bars denote the standard deviations calculated from three biological replicates (*P*<0.0001 using one-way ANOVA).

Evaluation of the expression of type II TA system genes in the presence of Ceftazidime (50X MIC) revealed an overall increase in expression for all studied genes (**Figure 14**), with *doc/phd* and *relE/relB* systems exhibiting the highest and *vapC/vapB* showing the lowest increase in expression. When examining gene expression in the presence of a combination of Ceftazidime with Nafcillin or Diosmin, a decrease in the expression of all genes compared to antibiotics alone was observed. These reductions were statistically significant (P<0.0001), except for *gnat* (P=0.99), *relE* (P=0.70), and *hha* (P=0.29) in the presence of Nafcillin and Ceftazidime, which did not significantly differ from Ceftazidime alone.

Similarly, analysis of gene expression in the presence of Ciprofloxacin (50X MIC) showed an increase in expression for all studied genes (**Figure 14**), with *gnat/rhh* systems exhibiting the highest and *doc/phd* showing the lowest increase in expression. When examining gene expression in the presence of a combination of Ciprofloxacin with Nafcillin or Diosmin, a decrease in gene expression compared to antibiotics alone was observed, with statistically significant reductions (P<0.0001), except for *relE2* (P=0.077) and *phd* (P=0.073) in the presence of Nafcillin and Ciprofloxacin, which did not significantly differ from Ciprofloxacin alone.

Moreover, evaluation of gene expression in the presence of Colistin (50X MIC) indicated an increase in expression for all studied genes (**Figure 14**), with *relE/parE* systems showing the highest and *hha/tomB* demonstrating the lowest increase in expression. When assessing gene expression in the presence of a combination of Colistin with Nafcillin or Diosmin, a decrease in gene expression compared to antibiotics alone was observed, with statistically significant reductions (P<0.0001), except for *relB* in the presence of Nafcillin and colistin (P=0.88), as well as Diosmin and Colistin (P=0.49), which did not significantly differ from Colistin alone.

## Discussion

*S*. Typhimurium is a Gram-negative bacterium that causes a significant burden of foodborne infections worldwide (Narimisa et al., 2021a). One of the key challenges in treating *Salmonella* infections is the presence of persister cells, which are able to survive antibiotic treatment and contribute to recurrent infections (Hill et al., 2021). The computational approach of repurposing drugs holds significant promise in unveiling novel applications for existing pharmaceuticals (Rudrapal et al., 2020). Herein, we explored the potential of using drug repositioning to identify Lon protease inhibitors in *S*.Typhimurium and investigated the effect of these inhibitors on the number of persister cells and the expression of type II TA genes.

Through screening a library of FDA-approved drugs for potential inhibitors of the Lon protease, our research identified Diosmin and Nafcillin as potent inhibitors of this protease. Diosmin, a well-known natural flavonoid utilized in the treatment of chronic venous insufficiency and varicose veins, has garnered attention for its various pharmacological properties, such as anti-inflammatory, antioxidant, and antibacterial effects (Zheng et al., 2020). Our study revealed that Diosmin displayed significant binding affinity to the Lon protease, with docking analysis demonstrating hydrogen bonds with Lys 722 and Ala 679 of the protease. Nafcillin, a narrow-spectrum beta-lactam antibiotic belonging to the penicillin class (Casto et al., 1979), exhibited high affinity for the Lon protease in our investigation, establishing a hydrogen bond with lysine 722. Additionally, we conducted molecular dynamics simulations to assess the stability of the Lon-Diosmin and Lon-Nafcillin complexes, with results indicating equilibrium within the complexes throughout the simulation period.

In this study, we conducted an investigation into the impact of various concentrations of Diosmin and Nafcillin on the number of viable cells. Our results demonstrate a noteworthy reduction in viable cell count when exposed to nafcillin at 64 μg/ml and 128 μg/ml, showing a significant decrease of 14.6, and 15.7 logarithmic units compared to the control group. Intriguingly, Diosmin concentrations of 100 µg/ml and 250 µg/ml did not exhibit significant effects on bacterial growth, akin to the control group. Conversely, at a concentration of 500 μg/ml, a remarkable decrease in viable cells was observed, resulting in a 5.5 logarithmic unit decrease compared to the control after 24 hours. The contrasting impacts of Nafcillin and Diosmin on bacterial growth underscore the significance of dosage and the distinct antimicrobial characteristics of each compound. The potent antibacterial effect of Nafcillin at higher concentrations may be attributed to its antibiotic properties. These discoveries enrich our comprehension of the antimicrobial mechanisms of Nafcillin and Diosmin, setting the stage for further exploration of their therapeutic potential in combating bacterial infections. We investigated the formation of persister cells in *S*. Typhimurium cultures when exposed to varying high concentrations of bactericidal antibiotics combined with Diosmin and Nafcillin. Combinations of Ceftazidime with either Nafcillin or Diosmin exhibited substantial reductions in bacterial cell counts, resulting in the elimination of viable cells within 24 hours. Furthermore, the combination of Ciprofloxacin with Nafcillin and Diosmin demonstrated high efficacy in decreasing bacterial cell numbers, along with a notable reduction in persister cells compared to Ciprofloxacin used alone. These results indicate promising potential for the synergistic effects of these combinations in addressing bacterial persistence. Our study revealed that combining Nafcillin or Diosmin with Colistin did not induce a significant decrease in live bacterial cells after 24 hours. Our findings suggest that the effectiveness of Nafcillin and Diosmin with various antibiotics can vary significantly, possibly due to the distinct mechanisms of action among different antibiotics and the activation of diverse pathways leading to persister cell formation.

Afterwards, we examined the expression of the Lon protease in *S*. Typhimurium when exposed to various antibiotics with the aim of advancing understanding regarding the potential impact of different antibiotics on enhancing protease expression. Our findings demonstrated that Ceftazidime and Ciprofloxacin notably influenced the activation of Lon protease, whereas Colistin exhibited a comparatively weaker effect. These findings align with the colony count results of the cells, suggesting that the presence of Colistin may have a lesser impact on the activation of Lon protease in the establishment of persister cells. Additionally, we evaluated the expression of type II TA systems after three hours of bacterial exposure to various antibiotics, including both individual antibiotics and a combination of Nafcillin and Diosmin. Consistent with previous researchs (Narimisa et al., 2020; Narimisa et al., 2021b), our study revealed distinct variations in the expression levels of different type II TA systems when exposed to various antibiotics. Furthermore, the analysis of the combination’s efficacy compared to antibiotics alone revealed the significant impact of Nafcillin and Diosmin in decreasing type II TA system gene expression. Although the reduction in gene expression of several genes was not significant in the presence of Nafcillin, Diosmin demonstrated superior efficacy in suppressing gene expression. This might be attributed to the higher number of hydrogen bonds, resulting in a more stable bond with Lon protease.

## Conclusion

This study highlights the potential of Nafcillin and Diosmin in targeting persister cells in *S*. Typhimurium infections. The findings of this study demonstrate the effects of these inhibitors on reducing persister cell populations and suppressing type II TA genes expression. These results can offer valuable insights into novel strategies for inhibiting bacterial persistence.

## Data Availability

The raw data supporting the conclusions of this article will be made available by the authors, without undue reservation.
